# Assessing the sustainable development and intensification potential of beef cattle production in Sumbawa, Indonesia, using a system dynamics approach

**DOI:** 10.1371/journal.pone.0183365

**Published:** 2017-08-17

**Authors:** Dahlanuddin, Benjamin Henderson, Kanar Dizyee, Hermansyah, Andrew Ash

**Affiliations:** 1 Faculty of Animal Science, The University of Mataram, Mataram, West Nusa Tenggara, Indonesia; 2 Commonwealth Scientific and Industrial Research Organization, Queensland Bioscience Precinct, St Lucia, Queensland, Australia; 3 UNE Business School, University of New England, Armidale, New South Wales, Australia; International Nutrition Inc, UNITED STATES

## Abstract

The intensification of beef cattle production in dryland areas of East Indonesia has the potential to substantially raise the incomes of smallholder farmers that dominate the sector. In this study we assess the potential for intensifying beef production on Sumbawa Island, by introducing a household feedlot production system (2–20 animals) based on the *Leucaena leucocephala* (leucanea) tree legume as an improved source of feed. We used a system dynamics approach to model the entire value chain, accounting for herd dynamics, demand dynamics and seasonality. Our findings complement the growing body of biophysical evidence about the potential success of this intervention, by simulating improvements in the annual profitability for beef farmers in the project area of up to 415% by 2023. Increases in farm profit were shown to depend near equally on the higher productivity of the leucaena feeding system and an associated price premium, demonstrating the importance of supporting improved agricultural production with better marketing practices. The intervention was also shown to generate positive or neutral benefits for the main post-farm value chain actors. Importantly, it also reduced the GHG emission intensity of outputs from the beef herd by 16% by 2020. We explored number of scale-out pathways, including a relatively moderate pace of autonomous adoption for our main analysis, resulting in the accumulation of 3,444 hectares of leucaena 20-years after the initial project phase, which could sustain the fattening of 37,124 male cattle per year. More ambitious rates of scale-out were found to be possible without exceeding the animal and land resources of the island.

## Introduction

The intensification of beef cattle production in dryland areas of East Indonesia has the potential to substantially raise the incomes of smallholder farmers that dominate the sector. With a growing body of research providing solid evidence about benefits of tree legume-based feeding systems for beef cattle production in this region [1], now is an opportune time to explore the potential for applying this intervention at a large scale. In this study we assess the potential for intensifying beef production on Sumbawa Island, Indonesia, using the *Leucaena leucocephala* (leucanea) tree legume as an improved source of feed [2] for the sector. Sumbawa Island is located in the province of Nusa Tengarra Barat (NTB). The beef cattle population of the island was 590,295 in 2015 and official statistics show it has been growing steadily over the past five years [3]. With high beef prices and growing demand for beef increasing sale and slaughter rates, official reports of an increasing cattle population are a subject of discussion and debate.

Cattle production in Sumbawa tends to be more extensive than other parts of Indonesia, typified by low input use and a reliance on poor quality roughages, including native grass and crop residues. This deficiency along with poor animal management results in low productivity and marginal economic returns. There are presently no commercial feedlots on the island, although a household fattening sector is beginning to emerge [4].

Most cattle, particularly fattened males, are exported live to Lombok and other markets outside NTB. In 2013 there were 24,526 slaughter cattle exported from Sumbawa Island to Lombok and 19,952 to markets outside NTB [4]. The cattle marketing system comprises numerous actors, including traders, brokers and butchers. Markets are reasonably competitive, although market power is quite concentrated in the final stages of the live export chain. However, a lack of formal price reporting and the use *ad hoc* judgements, rather than scales to measure animal weight, is a source of marketing inefficiency. While there is no clear evidence that intermediaries are exploiting this lack of transparency to extract excessive margins [4], the marketing system is limited in its capacity to reward farmers with higher prices for heavier and younger meat animals. As such, farmers are less likely to receive the financial returns and incentives they require to invest in intensifying production.

Existing research on beef production systems in Sumbawa, the neighbouring island of Lombok, and East Java provide a well-founded template for the intensification and development of the Sumbawa beef sector. Experimental results show that the weaned male Bali cattle fed luecaena hay have a daily live weight gain (LWG) of 0.47 kg/day [1], which is far superior to the 0.03 kg/day rate for animals fattened on native grass reported in that study. Other studies report similar improvements in performance, with male Bali cattle fed native grass found to have a LWG of around 0.1 kg/day [5] compared to rates of 0.22–0.42 kg/day [6] for cattle fed tree legumes. There is also solid evidence that these productivity improvements can translate into economic benefits, with improvements in cattle fattening profits of 172% possible through supplementing low quality animal diets with leucaena in East Java [7]. Further, beef fattening in Jati Sari (Sumbawa) with a leucaena based diet was found to generate net returns of 131,067 Indonesian Rupiah (Rp) per day per head in the wet season, compared to Rp 19,250 with a low-quality baseline diet [4].

However, on Lombok Island the potential for improved productivity at scale was limited by available land for growing forages [8]. Individual animal productivity and turn off rates could be dramatically increased but the overall cattle population did not increase in response to widespread adoption of improved nutritional and forage interventions because of land constraints. Human population is much lower on Sumbawa Island and land area per farmer is far greater, with much larger tracts of cleared extensive grazing land suited to leucaena production, which is not currently used for food crops.

The purpose of this research is to assess the potential for intensifying beef production on Sumbawa Island, by introducing a household feedlot production system (2–20 animals) based on feed from leucaena grown on-farm. The project site is part of the Applied Research and Innovation Systems in Agriculture (ARISA) project, which aims to increase smallholder incomes through the adoption of improved farming and value chain performance by brokering relationships between research institutes and the private sector (http://aip-rural.or.id/arisa/index.php/about).

We use a system dynamics (SD) approach to model the entire value chain, taking into account herd dynamics, demand dynamics and seasonality. We use this framework to assess the costs and benefits of livestock intensification, improved marketing efficiency and the opportunities and constraints for scaling up the intervention from an initial project site to the entire beef sector of the Sumbawa Island. Given the current starting point of low productivity and investment in the Sumbawa beef sector, such large scale change will be a substantial challenge and would depend on sustained extension support and sufficient market incentives to reward adoption [8]. Specific research questions include:

- What is the impact of the leucaena-based feedlot intervention on the net incomes of smallholders?
- How are these benefits shared among different value chain actors?
- How much additional gain can be obtained from demand-side measures that raise the price of cattle?
- What is the potential for scaling up the intervention, and to what extent are animal and land resources constraints likely to constrain this process?
- How large are environmental co-benefits of the intervention package in terms of lower greenhouse gas (GHG) emission intensities of beef products?

## Methods

### Data

The relevant data for this study were assembled at two different scales. Firstly, at the ARISA project site scale and secondly for the island of Sumbawa. The project area includes more than 70 farmer groups from the districts of Sumbawa and Sumbawa Barat, including 1,005 farms and a beef cattle population of 5,013. The cattle used in the project site and throughout Sumbawa Island are almost exclusively comprised of the Bali beef breed. Each farmer group contains 10 to 20 farmers that work together and sometimes share resources such as cattle pens and feed. The formation of these groups is encouraged by government to facilitate more efficient delivery of technical support. The baseline output of these cattle systems is low and annual sales of fattened male animals are only 351 head (hd) per year. Physical data, economic data and performance parameters for the breeding and fattening components of the project area beef herd are presented in Table 1. In the project feedlots, young male feeder stock are purchased at 140kg and after 154 days of feeding are sold at an average weight of 210 kg. This compares to 427 days for animals on a baseline diet and results in a shortening of the age at sale of from 3.3 to 2.5 years old. Cattle in the project feedlots are assumed to be fed a diet of 100% leucaena, equivalent on average to 5kg of dry matter (DM) per day, resulting in 770 kg of leucaena being required per animal over the 154 fattening period. Local research demonstrates the feasibility of fattening bulls on 100% leucaena diets without adverse effects on animal health [1]. It also makes economic sense because once the trees are established it more affordable than concentrate feed and there is a lack of good energy supplements available on farm. Given the average annual leaucaena yield of 8.3 tDM ha^-1^, each hectare of leucaena can support a feedlot throughput of 10.8 slaughter animals per year. By contrast, baseline animal diets are mainly comprised of native grasses occasionally supplemented with low quality crop residues such as maize leaves and rice straw.

**Table 1:** Baseline and project data for cattle breeding and fattening.

	Baseline	With project
**BREED HERD DATA & PARAMETERS**		
**Fertility rate (%)**	70	77^a^
**Calving interval (weeks)**	60	58^a^
**Age at first calving (years)**	4	4
**Natural abortion rate (%)**	5	5
**Calf mortality rate (%)**	10	10
**Heifer replacement rate (%)**	20	20
**FATTENING DATA & PARAMETERS**		
**Fattening duration with Leucaena (days)**	427	154
**Average purchase weight (kg)**	140	140
**Average sale weight (kg)**	210	210
**Average daily weight gain (kg day**^**-1**^**)**	0.16	0.45
**Dressing percentage (%)**	46–48	50–52
**Live weight cattle purchase price (Rp kg**^**-1**^**)**	37,000	37,000
**Live weight cattle sale price (Rp kg**^**-1**^**)**	37,000	42,550^b^
**Leucaena yield (t DM ha**^**-1**^**)**	n/a	8.3
**Cattle purchase price (Rp hd**^**-1**^**)**	5,180,000	5,180,000
**Cattle sale price (Rp hd**^**-1**^**)**	7,500,000	7,770,000 ^b^
**Leucaena establishment costs (Rp ha**^**-1**^**)**	n/a	1,689,030
**Feed collection costs (Rp hd**^**-1**^**)**	1,121,227	810,448
**Other animal costs (Rp hd**^**-1**^**)**	198,773	204,883

^a.^ These parameters are endogenously determined in the model simulation under Scenario 2 (described in the Scenario description and scope section). The values included in this table represent the estimated improvements 10 years from the commencement of the intervention.

^b.^ These prices reflect the 15% price premium for Scenario 2, described in the Scenario description and scope section.

*Source*: The live weight cattle price is taken from Waldron et al. [4] and all other costs were obtained through interviewing project staff and farmers (In compliance with PLOS ONE requirements for research involving human participants, we confirm that this project was approved by the CSIRO Social Science Human Research Ethics Committee).

The leucaena establishment costs include equipment for fencing, nursery needs (shading and poly bags), seeds and labour. The feed collection costs include both labour and motorbike fuel, and the other animal costs include veterinary costs, marketing costs, water and costs associated with the maintenance and construction of feedlots. In the simulations presented later, the costs and returns are based on year 2015 prices relevant to the study area. As shown in Table 2, the total cattle population for the island of Sumbawa is 590,295. This is the total population that will be considered for assessing different scenarios about scaling out the leucaena feeding intervention across the island of Sumbawa, as described in the Scenario description and scope section and the Results section.

**Table 2:** Cattle population data for Sumbawa Island in 2010–2015, by district.

	2010	2011	2012	2013	2014	2015
**Sumbawa Barat**	41,536	47,781	54,393	59,507	61,128	61,813
**Sumbawa**	156,797	162,924	197,141	215,675	216,167	228,826
**Dompu**	74,889	85,612	96,205	105,250	106,992	112,503
**Bima**	91,725	117,842	148,089	162,012	166,094	170,118
**Kota Bima**	16,781	12,034	13,592	14,870	15,180	17,035
**Total**	381,728	426,193	509,420	557,314	565,561	590,295

Source: Dinas Peternakan dan Kesehatan Hewan Provinsi NTB. Annual Report (2016).

### System dynamics model

We developed a dynamic simulation model, using an SD approach, to simulate the ex-ante impacts of our intensification, marketing and scale-out scenarios. We used iThink (http://www.iseesystems.com/) program to construct our model and the model structure and codes are available from the authors upon request. The full set of model equations and data are also provided in the supporting information for this paper (S1 Appendix). This modelling approach is grounded in control theory and the modern theory of nonlinear dynamics [9]. The SD approach incorporates dynamic interactions, feedback effects, and delays among different components of the system [9]. It is well suited to complex systems such as livestock value chains, in which time lags associated feed supply, breeding and fattening cycles, and the presence of market and resource scarcity feedbacks can generate complex and unintuitive system behaviour [10]. In recent years, several SD models have been used to simulate and assess the behaviour of livestock value chains over time. An SD model was also developed to assess the potential for the manufacturing and marketing of goat cheese in Mexico [11]. More recent studies used a SD approach to evaluate the commercialization goat value chains in Mozambique [12] and to evaluate the impacts of improved access to export markets in Namibia [13].

Each value chain sector and production process in our model is captured by a series of stocks and flows and their relationships and behaviour are modelled using differential and integral calculus. Examples of the main stocks in the model include the cattle population or herd and the area of land planted with leucaena. The cattle population is comprised of interlinked stocks animal cohorts, grouped on the basis of age, purpose and gender. These include breeding females, calves, weaners, males for fattening and for reproduction, and heifers for replacing breeding females and for sale as breeding stock on other islands.

These stocks of animals accumulate and decline over time according to the inflows and outflows to and from these stocks. Fertility, growth, mortality and cattle sales rates determine the size of these flows, and therefore the size of the stocks of cattle and land use over time. When in equilibrium, the inflows (births) and outflows (deaths and sales) balance out to maintain a steady population or stock of cattle. The baseline trajectory for the cattle population in our project area was gradually increasing, implying that inflows have been exceeding outflows over time in recent years.

The post-farm sectors of the value chain include inter Island traders who purchase and sell both heifers and fattened males, and local butchers who purchase and process cull animals for local consumption. Our model also introduces periodic demand shocks for three important Muslim festivals, namely, Eid Al Adha, Ramadan, and Prophet Muhammad’s Birthday (Mawlid). These events cause temporary, but relatively large spikes in consumption and cattle prices. We assume a 15% increase in the volume of male cattle consumed over the month of Ramadan, a 10% increase over one month for Mawlid, and a doubling in weekly rate of male cattle sold for slaughter over a two-week period in the lead up to Eid Al Adha. These spikes in demand are accompanied by a 10% price increase for Ramadan and Mawlid, and a 25% increase in the lead up to Eid Al Adha. We also capture the seasonality of breeding cycles in the farm system with conception mostly occurring at the beginning of the wet seasons and calving in occurring in the dry season.

**Fig 1:**
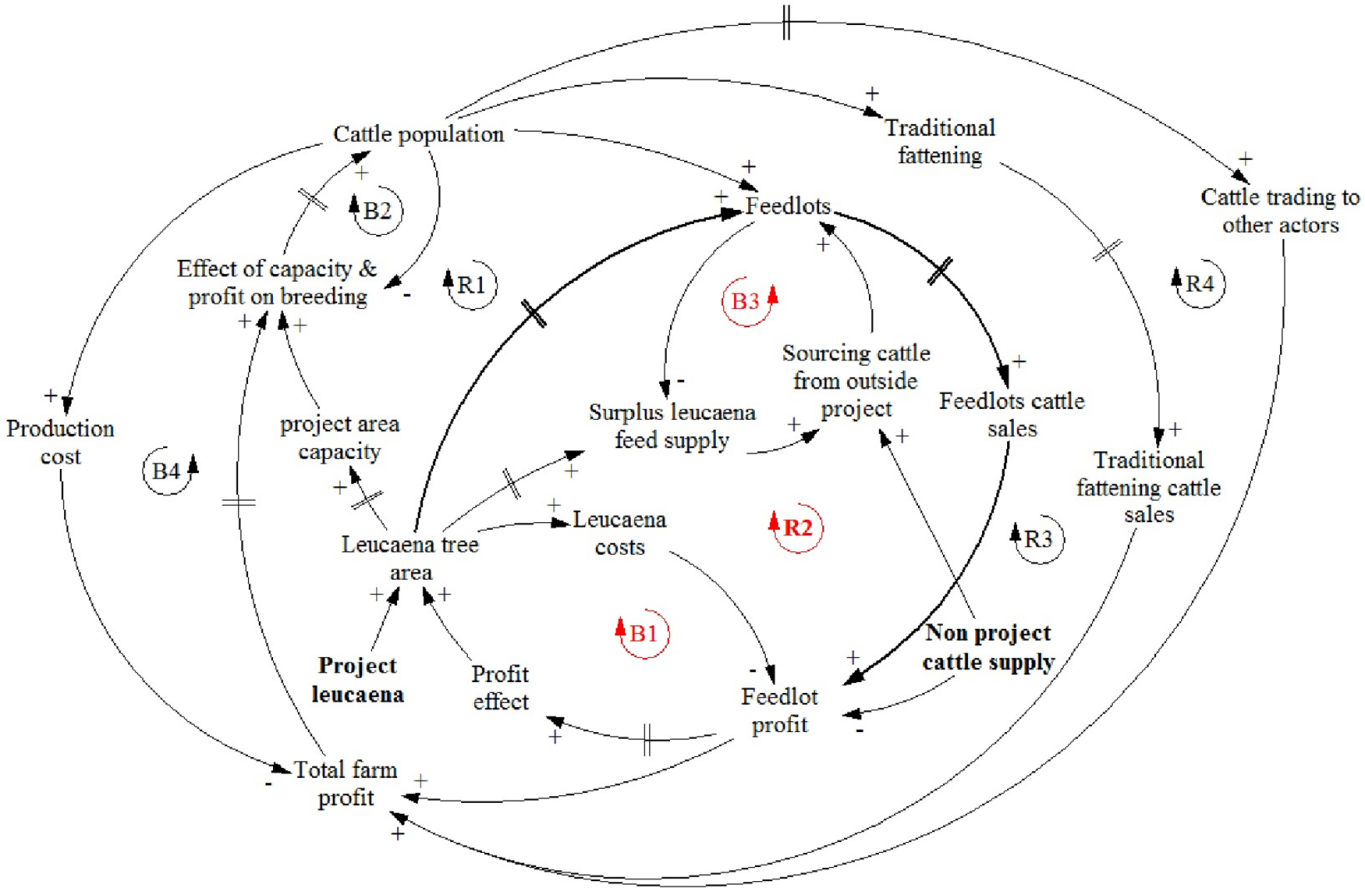
Portrait of model structure highlighting the main feedback loops in the model.

The male animal fattening component of our model is separated into traditional fattening and feedlot fattening subcomponents. Our feedlot intervention causes a shift of male feeder stock in the project area from traditional to feedlot fattening and a corresponding shift in the diets of male feeder stock from native grasses and other low quality forages to wild and planted leucaena. Feedlot enterprises also have access to male feeder stock from outside the project area, which is important for utilising feed from leucanea planted during both the project and scale-out phases of the intervention. Farm income is derived from the sale of males fattened for export, heifers exported for breeding, and from cows and bulls that are culled at the end of their service lives. In Fig 1, we provide a simplified causal loop diagram to summarise the model structure and highlight the major feedback loops in the beef sector in Sumbawa.

The arrows in Fig 1 represent the main causal relationships among the model components. The polarities (+ and − signs) on the arrowheads indicate the direction of these causal relationships. A positive (+) sign indicates that a change in a source variable will change the variable of destination in the same direction (e.g., an increase in variable ‘Feedlots cattle sales’ leads to an increase in ‘Feedlot profit’). In contrast, a minus (-) sign indicates the two correlated variables move in opposite directions (e.g., an increase in ‘Leucaena costs’ leads to a decrease in ‘Feedlot profit’, and vice versa).

There are a number of positive or reinforcing (R) and negative or balancing (B) feedback loops which regulate the overall dynamics of our value chain model. The symbol (//) denotes delays in the system (e.g., there is a time lag between change in ‘Feedlot profit’ and the decision to invest in leucaena (i.e. ‘Profit effect’), or the time it takes ‘Feedlots’ and ‘Traditional fattening’ operations to fatten cattle for sale. The primary positive feedback is the profitability of the cattle production system. Profit improvements, either through the feedlot or marketing interventions, encourage additional investments into leucaena planting and feedlot activity, and better animal husbandry which raises animal fertility (see R1 and R2 in Fig 1). On the other hand, the oversupply of leucaena tree areas increases leucanea costs which lowers feedlot profit and provides a negative feedback signal causing investment into leucaena planting to taper off (see B1). In a similar vein, an increase of surplus leucaena feed supply leads to an increase in the sourcing of cattle from outside the project area, which increases the cattle population in the feedlots and, hence, reduces surplus leucaena feed supply (See B3). The Sumbawa cattle supply chain system is governed by several additional feedback loops (as indicated in Fig 1), however, it is R2, B1, and B3 that are dominant feedback loops governing the model outcome during the scaling-out of the intervention. To explain the key relationship between leucaena plantings and feedlot profits in more detail, we display the following two model equations that govern this relationship:

$$Desired\;Leucaena\;Area_{t_{n+1}}=Profit\;Effect_{t_{n}}\;\times \;Leucaena\;Tree\;Area_{t_{n}}$$ (1)

$$Profit\;Effect_{t_{n}}=(Feedlot\;Profit{\;}_{t_{n}}/Feedlot\;Profit{\;}_{t_{0}}){\;}^{AE}$$ (2)

Where the desired future planting area ($Desired\;Leucaena\;Area_{t_{n+1}}$) is determined by the product of the existing area of mature trees ($Leucaena\;Tree\;Area_{t_{n}}$)and a variable that represents the impact of profit improvements on the desired planting area ($Profit\;Effect_{t_{n}}$), at time period t_n_. This variable is in turn determined by the profitability of feedlot production at any point in time ($Feedlot\;Profit{\;}_{t_{n}}$) relative to the profitability of feedlot production in the initial time period, t_0_, raised to the power of a parameter that can be described as the acreage elasticity (AE) with respect to profit. This parameter determines the responsiveness with which the land area planted with leucaena expands in response to an increase in profit. We assume a value of 0.15 in our analysis, which means that a 1% increase in profit will provide the incentive for a 0.15% expansion in the crop area. While acreage elasticity of supply with respect to prices have been estimated for many crops, very few studies have estimated acreage elasticities with respect to profit, and neither of these two parameters have been estimated for leucaena. We therefore based our selected AE value on a range of estimates for other crops. AE values varying between 0.16 and 0.33, depending on the time periods used, were estimated for aggregate cropland in Brazil [14]. The authors also translated these estimates to the more commonly reported acreage elasticities of supply with respect to price values of 0.38 and 0.90 [14]. Other relevant studies have estimated values for this parameter, with respect to price, of 0.77 for rubber plantations in Malaysia [15], and of 0 to 1.55 for a range of crops in the US [16]. Based on this sample of estimates in the literature, our assumed AE value of 0.15 can be considered to be relatively conservative. However, given the uncertainty about the value of this key parameter, we explore the sensitivity of the model results to a range of AE values from 0 to 0.3.

We also keep track of enteric methane emissions from the cattle herd over time. To do this we use the Tier 1 emission factor relevant for cattle from Asia, which is equal to 47 (kg CH_4_ head^-1^ yr^-1^), as well as Tier 2 emission factors which are more precise because they take into account the weights of different cattle cohorts within the herd (e.g. cows, heifers, bulls, replacement animals), their growth rates, and diet quality [17]. For the Tier 2 approach, we assume that the dry matter digestibility of fattening animals increases from 62%, in the baseline, to 67% [18] when fed with fresh leucaeana leaves in the project scenarios. To assess the GHG emission performance of the system we calculate emission intensities by dividing the methane emissions produced by each animal in the herd by the carcass weight (CW) of sales generated by the herd. The CW is calculated by multiplying the live weights of animals of animals exiting the beef herd by their dressing percentage. As shown in Table 1, the average dressing percentage of all animals sold in the baseline is assumed to be 47%, while the dressing percentage male animals fattened with leucaena is assumed to increase to 51%. We express emissions in carbon dioxide equivalent (CO_2_-eq) by multiplying methane emissions by its global warming potential of 25 for a 100-year time horizon [19].

### Scenario description and scope

There are two scenarios tested in addition to the baseline, which are summarised below:

**Scenario 1:** Conversion to a leucanea-based feedlot system
**Scenario 2:** Conversion to a leucanea-based feedlot system and marketing-based price improvement

For both scenarios, we assess the short-run impacts and long-run impacts. In the former we focus on a ten-year period, which is the time frame of most relevance to estimating the ARISA project impacts. To meet the ARISA project goal of increasing net farmer incomes by 30% by 2018, it is necessary to focus on measures that can deliver solid gains in the short-run. For the long run, we extend the time frame to between 25 and 40 years to assess the potential for scaling up the intervention from the project site to the entire island of Sumbawa.

The main impact from adopting the Scenario 1 intervention is an increase in the fattening rate of young male beef cattle. This requires the sourcing of animals from the beef herd within the project area and then from outside the project area, as the increase in the throughput of cattle in the project feedlots exhausts the stocks of locally sourced male cattle. As the intervention raises the profit of the system as a whole, we assume that farmers will also put additional effort into improving the performance of cattle breeding herds, to exploit the potential for higher returns and to increase the supply of feeder stock. As discussed in the Results section, this causes in an increase in the fertility rate and a reduction in the calving interval over time. In the initial project years, the potential and uptake of the feedlot enterprise is driven by scheduled plantings of leucaena. Following this, plantings are assumed to occur endogenously through diffusion and uptake in response to the success of the intervention, as higher profits encourage new plantings. In the first couple of years of the project, feedlot activity is low and reliant on the harvest of wild leucaena growing on common land until the new leucaena plantings mature.

Coupled with this, we assess the potential gains from marketing-based price improvements for slaughter animals implemented as a 15% price premium above the baseline market price. This is assumed to be achieved through two steps. Firstly, by working with trading companies to increase access to high valued markets, such as those in the hotel sector of Jakata, Mataram and other important urban centres. The greater reliability of the quantity of slaughter animals supplied and higher standardisation of quality in terms of attributes such as weight and age and higher dressing percentage, that are possible with the project intervention, should help facilitate the access to the higher value markets. In addition, we assume that more transparent marketing arrangements in determining prices, including more widespread use of scales by farm groups to measure animal weights and the provision of more current information on market price movements, will also underpin the price premium. While we have set the value of the premium to 15% somewhat arbitrarily, it is line with expectations based on anecdotal evidence about the price increases received by farmer groups that have improved their feeding systems, and adopted more transparent marketing arrangement.

## Results

The model results are presented at two different scales: firstly, short-run results are presented at the ARISA project site scale; and secondly long-run scale-out results are presented for the island of Sumbawa. Results from both Scenario 1 and Scenario 2 are presented in the short-run, however, for the purpose of brevity only Scenario 2 is presented in the section on the long-run results. Recall that the two scenarios are identical apart from the introduction of the price premium for feedlot fattened beef in Scenario 2. The implications of the leucaena-based feeding intervention on the GHG emission intensities of beef production are then presented in the final subsection.

### Short-run project scale

**Fig 2:**
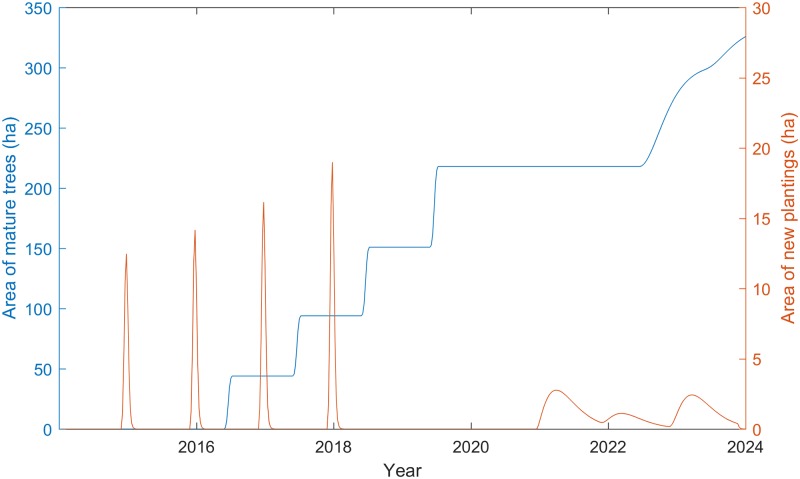
Scheduled and autonomous plantings of leucaena, and stocks of mature leucaena trees for Scenario 2.

In the initial project years, the potential and uptake of the feedlot enterprise is driven by scheduled plantings of leucaena (exogenous variable named *project leucaena* in Fig 1), which occur over the duration of the project (44, 50, 57, 67 ha yearly from 2015 to 2018). However, due to the time delay between planting and harvesting, the full benefits of these scheduled plantings are only realised in 2020 (Fig 2). At this point, there is some surplus in leacaena available for feed because of time lags between planting, trees maturing, and decisions about building feedlots and sourcing male feeder stock (see B3 feedback loop in Fig 1). As a consequence, there is a 17 month pause before the economic success of the intervention and the feed shortages combine to autonomously spur new plantings of leucaena (see R2 feedback loop in Fig 1). Feed from these additional trees becomes available after the maturation period of a further 18 months (delay mark // on the arrow between ‘leucaena tree area’ and ‘surplus leucaena feed supply’).

**Fig 3:**
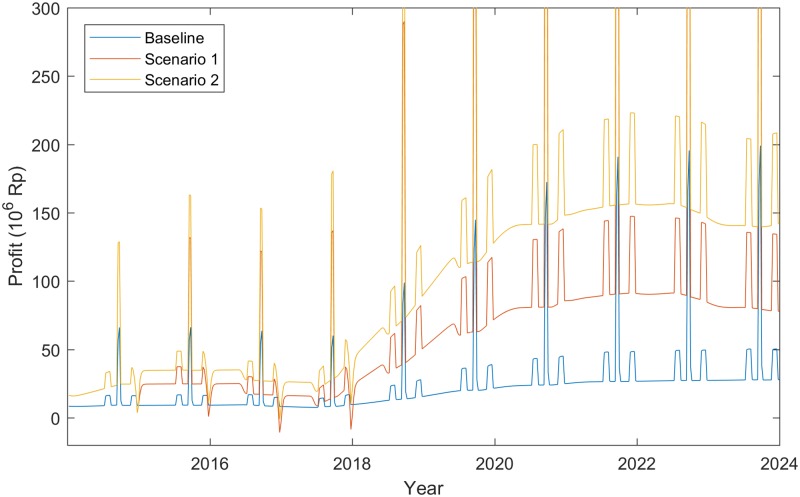
Weekly feedlot farm profits for the baseline, Scenario 1 and Scenario 2 (Rp. Per week).

The short-run economic success of the lecuaena feedlot intervention on cattle fattening profits is captured in Fig 3, where weekly farm profits from the intervention without (Scenario 1) and with (Scenario 2) the price premium are compared with the baseline situation. Note that the seasonal fluctuations in demand (due to celebrations around Idul Fitri, Eid al Adha and Mawlid) are clearly visible as spikes in profit. These are caused by a pulse in both the price of cattle and the volume of sales around these events (see Methods section). In the initial years of the project (2015 to 2018), the economic benefits from on farm plantings are are relatively low as the revenue from beef sales barely offset the costs of leucaena establishment. This is because of the relatively small area of planting and the time delay between planting and maturation of the trees. However, the use of wild leucaena in this initial period ensures that the new enterprise profits under Scenario 1 are 110% higher than in the baseline. By 2023, when both the project plantings have matured and the utilitization of leaucaena by cattle is close to complete, the increase in weekly profits in Scenario 1 surges to a level that is on average 220% higher than the baseline over the course of the year. With the addition of the Scenario 2 price premium, profits increase further, reach 415% of baseline levels in 2023. These results suggest that the returns of the price premium from improved marketing efficiency are of near equal importance to the gains that come from the adoption of the leucaena-based feedlot system. However, the former is not possible without the latter.

**Fig 4:**
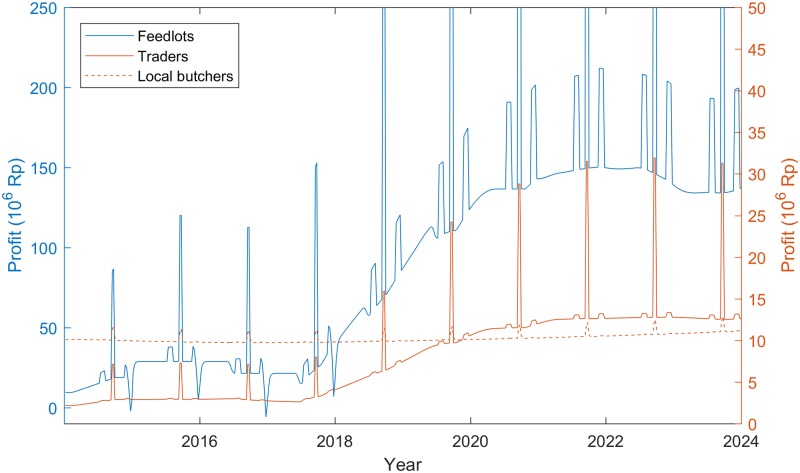
Weekly profits for the three main value chain actors for Scenario 2 (Rp. Per week).

The economic benefits of the intervention package extend beyond the farm gate, with varying impacts for different actors in the value chain (Fig 4). Unsurprisingly, the additional profits for traders track the pathway of profit flows for the feedlots, because the higher volume of throughput from these enterprises results in a higher volume of trades. Nevertheless, these benefits for the traders that source cattle from farmers adopting the package maybe offset, to a large extent, by losses to other traders that would have sourced fattened males that are now directed through the feedlots. In contrast to the farm sector, the project has no short-run impact on the throughput or profits of the local butcher sector. This is because this sector relies on cull animals from the breeding herd (which include older cows and bulls that reach the end of their service life), and the intervention does not increase the supply of these animals in the short-run.

### Long-run scale-out

In this section we present long-run simulation results to demonstrate the potential for scaling-out the leucaena-based feedlot intervention under the Scenario 2 package, which includes the 15% price premium from improved marketing. The key determinants for the long-run scale-out include the availability of male feeder stock, land for planting leucaena, and the responsiveness of new plantings to improvements in profit. According to local expert knowledge a ratio of weaned males available for fattening relative to the cattle population within the project area around 8–12% is likely depending on the performance of the breeding herd over time. Assuming a ratio 10%, this would result in 10% x 590,295 = 59,030 male cattle available for fattening within the island of Sumbawa in addition to the 351 available in the project area. Another factor that may constrain the scaling out of the intervention is the amount of land available for the planting of leucaena. However, given the high per hectare yield of leucaena and the relatively short fattening duration with this feed source, only 5,476 hectares would be required to supply the 59,030 annual throughput of male feeder stock (inclusive of project and non-project cattle) for 2015. To put this into context, there are 87,000 hectares of arable drylands in the Sumbawa district alone, much of which would be suited to leucaena production. Moreover, in the ARISA project area leucaena is being planted in hilly fallow areas not suited to other crops and is therefore unlikely to be attracting opportunity costs related to the displacement of other crops.

**Fig 5:**
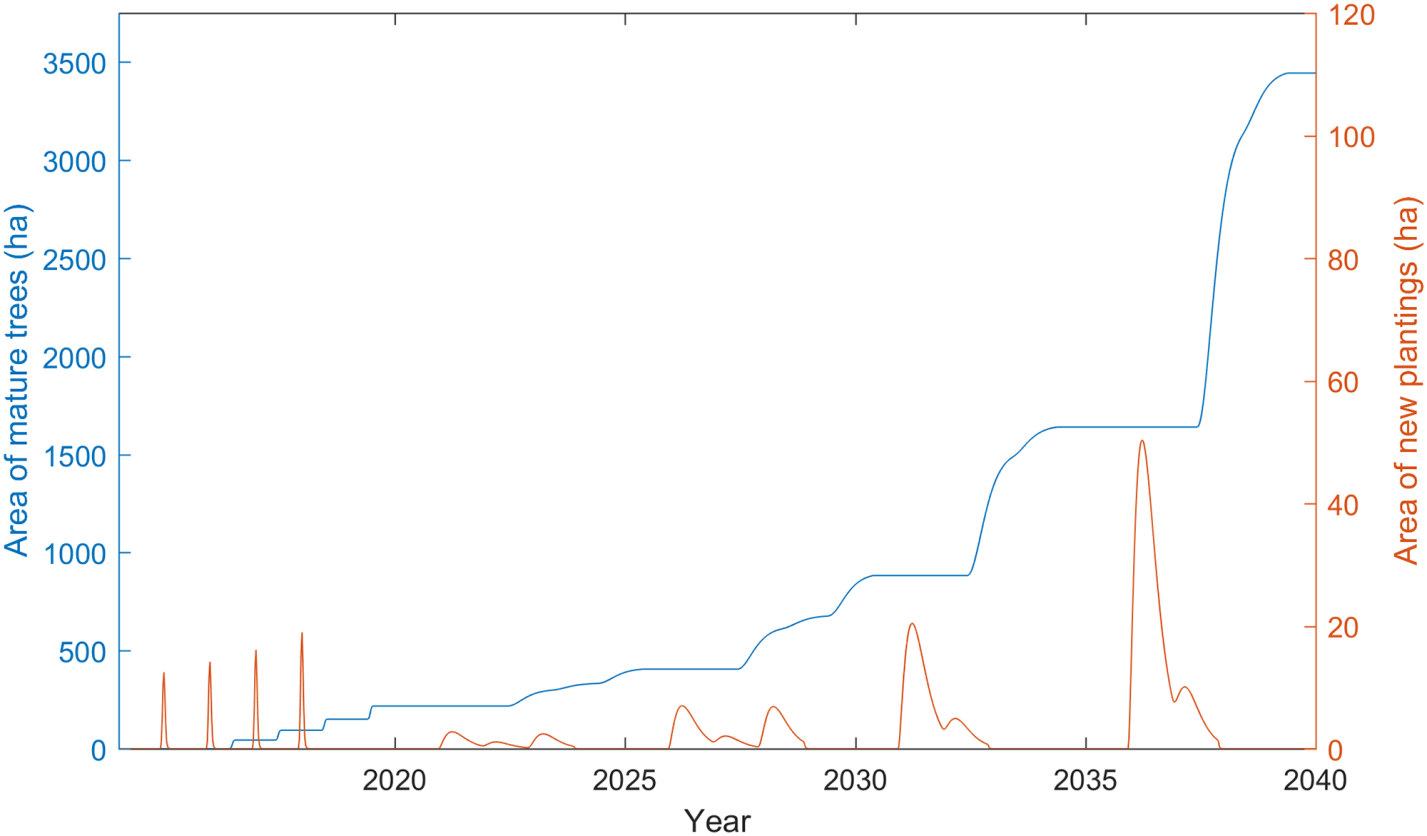
New plantings and corresponding stock of mature leucaena trees in the long-run for Scenario 2.

Under these constraints and model assumptions, the project scale-out proceeds at a pace of 19% per year from the time that project plantings reach full maturity and new endogenous plantings commence at the beginning of 2021 until the end of 2028. This amounts to an average of 83 additional hectares of trees each year, which is close to double the average rate of 42 hectares per year in the planting rate during the project phase from 2015 to 2019. After this the pace of scale out accelerates, with an average annual increase of 364 ha in leucaena planted between 2031 and 2038. The total stock of mature leucaena plantings reaches 3,444 ha by mid-2039 (Fig 5). By this time the corresponding throughput of fattened males reaches 37,124 head per year, which represents 63% of the total annual availability of 59,030 male feeder stock on the island of Sumbawa.

A notable feature of long-run scale-out is the cyclical nature of the autonomous leucaena plantings over time (governed mainly by R2, B1, and B3 feedback loops in Fig 1), with sowing events occurring over four separate oscillations ranging from 2 to 3 years in duration (Fig 5). These planting events increase in amplitude over time, but the area planted in each event comprises a similar share of the stock of mature trees in each corresponding time period. Such oscillations are typical in all economic industries and occur as a consequence of time delays in information feedbacks within production and value chain systems [9]. In our case, the decision to plant leucaena is motivated by shortages in the supply of leucaena relative to the amount of cattle available to fatten and profitability of feedlot enterprise. However, the decision to begin and cease planting is imperfect because of time delays in receiving and acting upon information about shortages and excesses in supply and forming an expectation of future profit of cattle production. The resulting periods of oversupply and undersupply causes the oscillating behaviour of leucaena planting.

From the perspective of land and animal resource availability on the island, the scale-out pathway displayed in Fig 5 proceeds at a relatively moderate pace. Since the throughput of fattened male animals corresponding to the maximum area of planted leucaena accounts for less than two thirds of the volume of male feeder stock available each year on the island, it is possible that both the pace and total size of scale-out could be increased. Moreover, the area sown to lecuaena represents a mere fraction of the potential area that could support this forage crop (see Data section). Since animal and land resources are not binding, we next turn our attention to adjusting the responsiveness of the intervention scale-out to profit improvement. As discussed, this responsiveness is driven by the (AE) acreage parameter.

The purpose of this analysis is not to construct a definitive or accurate projection. It is instead intended to identify which production resources could constrain the scaling-out of the intervention under differing but plausible rates of expansion. To explore these resource-based boundaries we assess the sensitivity of the results to a range of AE values. The yellow line (AE = 0.15) in S1 Fig corresponds to the value assumed in the preceding analysis and provides a useful reference point for the other scale-out pathways. At one extreme, an AE value to 0.05 results in very little autonomous plantings over the 35-year timeframe. At the other extreme an AE value of 0.3, which is not implausible, results in a rapid scale-out with the area of plantings reaching a maximum of 5,430 ha, by 2033. According to the model outputs, this area of leucaena supports annual throughput of 58,836 fattened males, which represents 99.7% of the total number of male feeder stock available each year. Hence, at this level of responsiveness, the intervention system can fully exploit all the animal resources available on the island of Sumbawa. In contrast, the land resources needed to support 5,430 ha of leucaena are again highly unlikely to constrain a scale-out of this size. There are however a number of reasons, which are articulated in the Discussion, why this level of scale-out may be difficult to achieve in practice. In addition to the sensitivity analysis that was performed as part of the scale-out assessment, we performed a number of validation tests recommended for SD models [9, 20], for which the model performed well (See S2 Appendix).

### Greenhouse gas emissions

The increase in the productivity of the cattle herd that is possible with the improved leucaena-based feedlot enterprise also has important implications for its environmental performance. We assess this by comparing the GHG emission intensities of production for the herd as a whole using both Tier 1 and Tier 2 emission accounting approaches for enteric methane in 2020 (Table 3). The same percentage reduction is estimated with both approaches, however the Tier 1 approach overestimates the emission intensities for both the baseline and for the project scenario. This overestimation occurs because the cattle in Sumbawa are smaller than the average cattle in Asia upon which the Tier 1 emission factor is based. The improved environmental performance in scenario 2 is mainly driven by a reduction in the lifespan of the male fattening animals, which reduces the overall stock of animals and their associated emissions required to support any given level of output. It is also improved, to a lesser extent, by the higher dressing percentage of the fattened animals (Table 1) and, in the case of the Tier 2 approach, from higher digestibility of feed for fattened animals.

**Table 3:** The GHG emission intensity of production (kgCO_2_-eq kg CW^-1^) with regard to enteric methane production in 2020.

	Baseline	Scenario 2	Reduction
**Tier 1**^a^	60.9	51.2	16%
**Tier 2** ^b^	45.2	38.2	16%

^a.^ These results are based on the IPCC Tier 1 emission factor relevant for cattle from Asia, which is equal to 47 (kg CH_4_ head^-1^ yr^-1^) [17].

^b.^ These results are based on IPCC Tier 2 emission factors, which are calculated by taking into account the weights of different cattle cohorts within the herd (e.g. cows, heifers, bulls, replacement animals) and performance factors including growth rates and diet quality [17].

## Discussion

In simulating the adoption of a leucaena-based feedlot fattening system in Sumbawa, we have shed light on the potential economic gains of the new system, its varying impacts on different value chain actors and its potential for improving environmental performance. In this section we summarise these findings and discuss how they can assist in improving project design in the short-run and in understanding the possibilities and constraints associated with various scale-out pathways in the long-run.

Our findings complement the growing body of biophysical evidence about the potential success of this intervention in Eastern Indonesia [1, 5, 6], by estimating improvements in the annual profitability for beef farmers in the ARISA project area of up to 415% by 2023. Due to the time it takes for scheduled plantings to reach completion and mature, the benefits of the intervention package also take some time to manifest. Therefore, investors and policy makers need to be patient and work within a sufficiently long-term planning horizon. Increases in farm profit were shown to be near equally dependent on adoption of the feedlot system and the price premium components of the package. This demonstrates the importance of incorporating both components. Importantly, the intervention was shown to generate positive or neutral benefits for the main value chain actors, although it will lead to a rearrangement of farmer-trader relationships as the intervention is scaled-out, particularly if price premiums can be secured and sustained for feedlot fed slaughter animals.

There are also climate change mitigation benefits from the intervention, with reductions in the GHG emission intensity of meat produced by the entire beef herd of 16% in 2020. This is low compared to the 20–57% reductions in emission intensity associated with switching from native grasses to leucaena reported for northern Australian beef systems [21, 22], because leucaena feeding was confined to the male fattening component of the herd in the present study. The baseline emission intensity estimated in this study (45 kgCO_2_-eq kg CW^-1^) is also relatively high compared to studies in other regions. For example, the FAO report Tier 2 emission intensities for beef production of around 28 kgCO_2_-eq kg CW^-1^, for enteric methane emissions in East and South East Asia combined [23]. Our higher estimate reflects the low level of herd productivity in Sumbawa, especially compared to production in East Asia. The constrast is even greater in some other developed regions. For instance, enteric methane emission intensities of 8 kgCO_2_-eq kg per kg of live weight (approximately 16 kgCO_2_-eq kg CW^-1^) are reported in northern Australia [21]and 7 kgCO_2_-eq kg per kg of live weight (approximately 14 kgCO_2_-eq kg CW^-1^) in central France [24]. These studies also show that enteric methane is dominant, comprising between 82% [24] and 95% [21] of animal GHG emissions, with the rest from manure management and deposition on pasture.

In addition to these substantial gaps in emission intensities, beef productivity in Sumbawa is also relatively low. Recall that the ADGs for fattening animals in our study area in the baseline and with leucaena feeding were 0.16 and 0.45 kg day^-1^, respectively. Even the improved rate of fattening in our project falls short of what is typically observed with heavier breeds in more developed regions. For example, ADGs of between 0.6 and 0.7 kg day^-1^ were reported for Aberdeen Angus steers finished on grass of in New Zealand [Aberdeen Angus steers finished on grass],with ADGs of up to 1.66 kg day^-1^ for the same breed finished on concentrate feed in France [25]. In China, local cattle breeds fed a mixture of straw and concentrates in typical Chinese production system had ADGs of between 0.78 and 0.82 kg day^-1^, while a rate of 1.5 kg day^-1^ was reported for imported Limousin cattle [26]. Cross breeds have also been found to perform better than local breeds in China, reaching heavier slaughter weights at a younger age [27]. The introduction of new genetics, possibly through cross breeding local and imported cattle, may provide additional gains to better feeding and animal management in Sumbawa. However, according to local experts, a previous attempt in the province of West Nusa Tenggara to introduce Brahman cross cows did not succeed, because farmers lacked the skills to manage larger breeds and did not have sufficient feed resources to make use of the animals’ higher potential. The Indonesia Australia Commercial Cattle Breeding Program (IACCB) is a pilot program launched in early 2016 that aims to overcome these challenges and assist in commercialising beef production in Indonesia through a package of measures that includes introducing Braham cattle and improving resource utilisation (www.iaccbp.org). The success of this program in overcoming previous obstacles to breed improvement will only be known once it has been completed and evaluated.

We also explored number of pathways to scale-out in the long-run, based on differing assumptions about the responsiveness of leucaena planting in response to higher economic returns for farmers. This responsiveness is a matter of some conjecture for which we assume a relatively moderate and realistic pace of autonomous scale out, resulting in the accumulation of 3,444 hectares 20-years after the initial project phase, which could sustain the fattening of 37,124 male cattle per year. This level of throughput represents 63% of the entire male feeder stock on the island of Sumbawa. Whether this pace of scale-out could be sustained over this timeframe on a purely autonomous basis, without being helped along by additional government investments and programs is not entirely clear. However, from the perspective of animal and land resource availability, higher rates of scale-out are certainly possible and under more optimistic assumptions could reach a scale capable of utilising virtually all of the 59,030 available male feeder stock on Sumabawa Island each year, some 15 years from the completion of the ARISA project. This result contrasts with those of another study on neighbouring Lombok, where land constraints limited the scale out of a package of interventions to improve cattle productivity [8].

While land resource availability is unlikely to constrain this level of production in Sumbawa there are a number of reasons why a burgeoning feedlot sector might face difficulties in securing all of the island’s male feeder stock over this time frame. Some of these constraints, based on feedback from a number of project sites across Indonesia in which leucaena-based feeding interventions have been introduced, are summarised in [28]. They found that some farmers prefer open grazing to cut-and-carry feeding, because of difficulties in meeting the much higher additional labour requirements of the latter system. Other reasons why farmers may resist adopting the new system include perceived higher risks of animal theft in the more built-up areas that feedlots tend to be located as well as a lack of capacity in tree establishment and access to seeds [27].

In some respects, the more moderate rate of scale-out in our main analysis (Fig 4) will reflect some of these additional constraints. However, this and more rapid rates of expansion are highly dependent on farmers receiving a price premium for younger, higher quality, fattened animals. Our results show that this marketing improvement nearly doubles smallholder profits from the intervention and is therefore integral to the scalability of the overall package. The widespread uptake of beef intensification practices, including the use of tree legume forage, is only likely to possible when coupled to such market-based incentives [8]. Supporting improvements in agricultural production with better marketing outcomes is key objective of the ARISA project, and understandably so given how fundamental it is to driving and sustaining the innovation process.

## Supporting information

S1 Fig Stocks of mature planted leucaena trees in the long-run for Scenario 2 under the different acreage elasticities of supply (0.05, 0.1, 0.15, 0.2, 0.3). (TIF)

S1 Appendix Full list of model equations and data. (DOCX)

S2 Appendix Model validation tests. (DOCX)
