# Supplementary figures and images for: Assessing the sustainable development and intensification potential of beef cattle production in Sumbawa, Indonesia, using a system dynamics approach
Source: PLoS One. 2017 Aug 17;12(8):e0183365. doi: 10.1371/journal.pone.0183365 (PMC5560717; doi:10.1371/journal.pone.0183365)

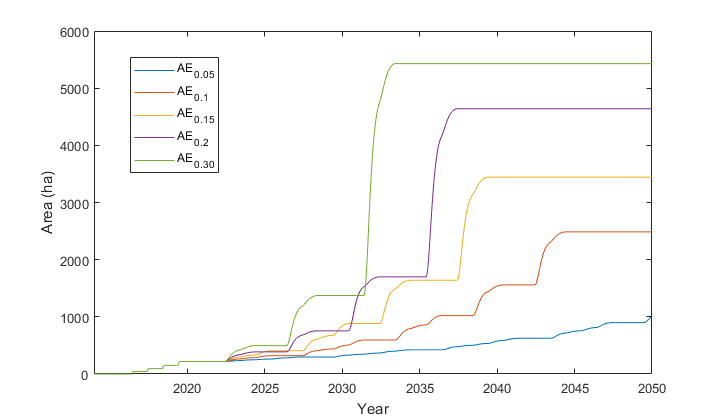

Supplement: S1 Fig — (TIF) [file pone.0183365.s001.tif]
