# Supplementary material for: Assessing the sustainable development and intensification potential of beef cattle production in Sumbawa, Indonesia, using a system dynamics approach
Source: PLoS One. 2017 Aug 17;12(8):e0183365. doi: 10.1371/journal.pone.0183365 (PMC5560717; doi:10.1371/journal.pone.0183365)
Supplement: S1 Appendix — (DOCX) [file pone.0183365.s002.docx]

**S1 APPENDIX. Full list of model equations**

This document contains all of the equations and data used to create and run the system dynamics model in iThink for the study: “Assessing the sustainable development and intensification of beef cattle production in Sumbawa, Indonesia, using a system dynamics approach”. The equations are presented in standard iThink/Stella format.

**Herd module**

**1. Cattle in gestation**

Gestation(t) = Gestation(t - 1) + (Breeding rate - Birth rate - Aborting)

INIT Gestation = 780

{cattle}

INFLOWS:

Breeding rate = ((Breeding cows*Stock 1)/8)*Actual effect of capacity breeding

{cattle/week}

OUTFLOWS:

Birth rate = Gestation/gest time

{cattle/week}

Aborting = (Gestation*abortion rate)/52

{cattle/week}

Abortion rate = 0.05

{1/year}

Actual effect of capacity breeding = MIN((SMTH3(Effect of capacity on breeding*Actual effect of profit on production,12)),1.6666)

{unitless}

Graphical function (Effect of capacity on breeding):

Cattle to capacity ratio = (cattle population-feedlot fat-Trad fat)/Project area capacity

{unitless}

Project area capacity = 8000+Local capacity changes

{cattle}

Local capacity changes = SMTH3(Surplus feed CE, 104, 0)

{cattle}

Actual effect of profit on production = SMTH3(Effect of profit on cattle production,26)

{unitless}

Effect of profit on cattle production = (Total farm profit/init(Total farm profit))^0.1

{unitless}

**2.**  **Changes to stock of calves**

Calves(t) = Calves(t - 1) + (Birth rate - weaning - Calves dying)

INIT Calves = 580

{cattle}

INFLOWS:

Birth rate = Gestation/gest time

{cattle/week}

Gest time = 39

{weeks}

OUTFLOWS:

Weaning = Cal2ves/Weaning time

{cattle/week}

Calves dying = (Calves*calf mortality rate)/52

{cattle/week}

Calf mortality rate = 0.1

{1/year}

Weaning time = 40

{week}

**3. Changes to stock of weaners**

Weaners(t) = Weaners(t - 1) + (weaning - Becoming young adult male - Becoming young adult female)

INIT Weaners = 184

{cattle}

INFLOWS:

Weaning = Calves/Weaning time

{cattle/week}

OUTFLOWS:

Becoming young adult male = (Weaners/time to grow)*Male to female ratio

{cattle/week}

Becoming young adult female = (Weaners/time to grow)*Male to female ratio

{cattle/week}

Male to female ratio = 0.5

{unitless}

Time to grow = 20

{week}

**4. Changes to stock of pre-adult females**

Pre adult female(t) = Pre adult female(t-1) + (Becoming young adult female - Becoming Heifer)

INIT Pre adult female = 238

{cattle}

INFLOWS:

Becoming Y female = Becoming young adult female*percent breeders to II traders

{cattle/week}

OUTFLOWS:

Becoming Heifer = Pre adult female/Growth time

{Cattle/week}

Percent breeders to II traders = 0.2

{unitless}

Growth time = 90

{weeks}

**5. Changes to stock of heifers for selling to breeders**

Heifer(t) = Heifer(t-1) + (Becoming Heifer - selling breeders to II traders)

INIT Heifer = Becoming Heifer

{cattle}

INFLOWS:

Becoming Heifer = Pre adult female/Growth time

{cattle/week}

OUTFLOWS:

Selling breeders to II traders = Heifer*1

{cattle/week}

**6. Changes to stock of females to replace breeding cows**

Replace female(t) = Replace female(t - 1) + (Becoming replacement female - Becoming breeding cows)

INIT Replace female = 1009

{cattle}

INFLOWS:

Becoming replacement female = Becoming young adult female*(1-percent breeders to II traders)

{cattle/week}

OUTFLOWS:

Becoming breeding cows = Replace female/Time to become breeding cows

{cattle/week}

Time to become breeding cows = 27+120

{week}

**7.**  **Changes to stock of breeding cows**

Breeding cows(t) = Breeding cows(t - 1) + (Becoming breeding cows - breed cow selling to butcher)

INIT Breeding cows = 1874

{cattle}

INFLOWS:

Becoming breeding cows = Replace female/Time to become breeding cows

{cattle/week}

OUTFLOWS:

Breed cow selling to butcher = Breeding cows/Average breeding duration

{cattle/week}

Average breeding duration = 273

{week}

**8. Changes to stock of males to replace breeding bulls**

Replace bull(t) = Replace bull(t-1) + (Becoming replacement bull - Becoming bull)

INIT Replace bull = 140

{cattle}

INFLOWS:

Becoming replacement bull = (Becoming young adult male*percent for replacing bulls)

{cattle/week}

OUTFLOWS:

Becoming bull = Replace bull/Time to become bull

{cattle/week}

Percent for replacing bulls = 0.1912

{unitless}

Time to become bull = 47+30

{week}

**9.**  **Changes to stock of breeding bulls**

Bull(t) = Bull(t - 1) + (Becoming bull - Bull selling)

INIT Bull = 165

{cattle}

INFLOWS:

Becoming bull = Replace bull/Time to become bull

{cattle/week}

OUTFLOWS:

Bull selling = Bull/Bull service time

{cattle/week}

Bull service time = 91*Ramadan & Adha Shock*Maulud demand shock

{week}

**10. Changes to stock of traditionally fattened bulls**

Trad fat(t) = Trad fat(t - 1) + (Males for traditional fattening - Trad fat sales)

INIT Trad fat = 881.21

{cattle}

INFLOWS:

Males for traditional fattening = (Becoming young adult male*percent remain in traditional farms M)

{cattle/week}

OUTFLOWS:

Trad fat sales = Trad fat/Grazing time M

{cattle / week}

Grazing time M = 130*Ramadan & Adha Shock

{week}

Percent remain in traditional farms M = 0.81-percent moving to feedlots

{unitless}

**11. Changes to stock of young pre-feedlot males**

Pre FL male(t) = pre FL male(t-1) + (backgrounding & feedlotting - back grounding)

INIT pre FL male = 63

{cattle}

INFLOWS:

Backgrounding & feedlotting = (Becoming young adult male*percent moving to feedlots)

{cattle / week}

OUTFLOWS:

Backgrounding = delay(backgrounding & feedlotting, backgrounding time)

{cattle / week}

Backgrounding time = 69

{weeks}

Percent moving to feedlots = MAX((MIN((Surplus feed CE/Becoming young adult male), 0.9)), 0)

{unitless}

**12. Changes to stock of feedlot fattened cattle (including project farm (PF) and non PF, young adult males (YAM)**

Feedlot fat(t) = feedlot fat(t - 1) + (back grounding + additional flows through PF feedlots - PF FL sales)

INIT feedlot fat = 100*policy switch 1: shift to FL

{cattle}

INFLOWS:

Back grounding = delay(backgrounding & feedlotting, backgrounding time)

{cattle / week}

Policy switch 1: shift to FL = [0 or 1]

{unitless}

Additional flows through PF feedlots = (Non PF YAM inputs*percent to feedlots)

{cattle / week}

OUTFLOWS:

PF FL sales = feedlot fat/fattening time

{cattle / week}

fattening time = 22*Ramadan & Adha Shock*Maulud demand shock

{weeks}

Percent to feedlots = DELAYn(1,4,3, 0)*policy switch 2: source nPF bulls

{unitless}

Policy switch 2: source nPF bulls = [0 or 1]

{unitless}

**13. Functions governing the timing of breeding events**

Breeding season(t) = Breeding season(t - 1) + (Start B – End B)

INIT Stock 1 = 0

{unitless}

INFLOWS:

Start B = breed week

{unitless/week}

OUTFLOWS:

End B = DELAY(Start B, 8)

{unitless/week}

Breed week = pulse(0.6,30, 52 )

{unitless/week}

**Young adult males sourced from outside the ARISA project area**

YAMs from non PFs(t) = YAMs from non PFs(t - 1) + (Non PF YAM inputs - additional flows through PF feedlots - exits to Non PFs)

INIT YAMs from non PFs = 15015

{cattle}

INFLOWS:

Non PF YAM inputs = MIN((MM supply shortage*Cattle shortage fulfilment ratio), Regional supply YAM)

{cattle / week}

OUTFLOWS:

Additional flows through PF feedlots = (Non PF YAM inputs*percent to feedlots)

{cattle / week}

MM supply shortage = MAX((Surplus feed CE-backgrounding & feedlotting), 0)+PF FL sales

{cattle/week}

Exits to Non PFs = Non PF YAM inputs*(1-percent to feedlots)

{cattle/week}

Regional supply YAM = ((590295-5013)*0.1)/52

{cattle/week}

**Cattle sale volumes and profits**

Flow of animals to II traders = selling breeders to II traders+ Selling traditional fatteners to II traders M+ Bull selling/2+selling FL fatteners to II traders

{cattle / week}

Flow of animals to local butchers = breed cow selling to butcher+ Bull selling/2

{cattle/week}

Total FL sales = PF FL sales

{cattle/week}

YAMexits = Males for traditional fattening+backgrounding & feedlotting

{cattle / week}

Trad fattening profit = Trad fat sales*(trad fat price per hd-cost per hd)

{Rp / week}

Trad fat price per hd = 7770000*Effect of shocks on price

{Rp / cattle}

Cost per hd = 6500000

{Rp/cattle}

FL profits = Total FL sales*(price per hd-other costs per hd) - leucaena costs

{Rp / week}

Price per hd = (if Petahani trade policy=1 and time > 52 then 7770000*1.15 else 7770000)*Effect of shocks on price

{Rp/cattle}

Petahani trade policy = [0 or 1]

{unitless}

Other costs per hd = 5384883

{Rp/cattle}

leucaena costs = Planted leucaena area mature trees*168000

+Leucaena over time*1689030

{Rp/week}

Breed farm profit = (breed cow selling to butcher*cow price+ Bull selling*bull price+ selling breeders to II traders*RFexit price+YAMexits*YAM price)-breed cattle population*cost per breed animal

{Rp/week}

Cow price = 7800000

{Rp/cattle}

Bull price = 11,820,000*Effect of shocks on price

{Rp/cattle}

YAM price = 5180000

{Rp / cattle}

RFexit price = 6300000

{Rp/cattle}

Cost per breed animal = 15000

{Rp / cattle / week}

Total farm profit = breed farm profit+ trad fattening profit+ FL profits

{Rp/week}

Trader profits = Total FL sales*sale price+Trad fat sales*sale price-Trader costs

{Rp/week}

Purchase Price per hd = 7770000

{Rp/cattle}

Trader costs = Trad fat sales*(purchase Price per hd+ Marketing costs)+Total FL sales*(purchase Price per hd+ Marketing costs)

{Rp/week}

Marketing costs = 333333

{Rp/cattle}

Sale price = 8300000

{Rp / cattle}

Local butcher profits = (Bull selling/2+breed cow selling to butcher)*profit per hd

{Rp / week}

profit per hd = 1300000

{Rp / cattle}

**Cattle population**

Breed cattle population = cattle population-feedlot fat-Trad fat

{cattle}

Cattle population = Weaners+ Calves+ Pre adult female+ Trad fat+ Heifer+ Replace bull+ Bull+ Replace female+ feedlot fat+ Breeding cows

{cattle}

Domestic population = (cattle population-feedlot fat) + pre FL male

{cattle}

Project fatten pop = Trad fat+ feedlot fat+ pre FL male

{cattle}

**Consumption shocks for Muslim festivals**

Eid Shock(t) = Eid Shock(t - 1) + (A Start - A End)

INIT Eid Shock = 0

{unitless}

INFLOWS:

A Start = Eid al Adha

{unitless/week}

OUTFLOWS:

A End = DELAY(A Start, 2)

{unitless/week}

Eid al Adha = pulse(1, 36, 52)

{unitless/week}

Maulud Shock(t) = Maulud Shock(t) + (M Starts - M Ends)

INIT Maulud Shock = 0

{unitless}

INFLOWS:

M Starts = Prophet Mohamad birthday

{unitless/week}

OUTFLOWS:

M Ends = DELAY(M Starts, 4)

{unitless/week}

Prophet Mohamad birthday = pulse(0.05, 46, 52)

{unitless/week}

Maulud demand shock = (if Maulud Shock>0 then 0.9524 else 1)

{unitless}

Ramadan Shock(t) = Ramadan Shock(t-1) + (R Start - R End)

INIT Ramadan Shock = 0

{unitless}

INFLOWS:

R Start = Ramadan holy month

{unitless/week}

OUTFLOWS:

R End = DELAY(R Start, 4)

{unitless/week}

Ramadan holy month = PULSE(0.0375, 27, 52)

{unitless/week}

Ramadan & Adha Shock = (if Eid Shock>0 then 0.4 else 1)+ (if Ramadan Shock>0 then 0.964 else 1 )-1

{unitless}

Effect of shocks on price = (if Maulud Shock or Ramadan Shock >0 then 0.1 else 0) + (if Eid Shock>0 then 0.25 else 0)+1

{unitless}

**Leucaena production**

Leucaena over time(t) = Leucaena over time(t-1) + (Input - Output)

INIT Leucaena over time = 0

{ha/week}

INFLOWS:

Input = Land allocation

{ha/week}

OUTFLOWS:

Output = Leucaena over time

{ha/week}

Planted leucaena area mature trees(t) = Planted leucaena area mature trees(t-1) + (Maturing)

INIT Planted leucaena area mature trees = 0

{ha}

INFLOWS:

Maturing = delay(Land allocation, Time to mature)

{ha/week}

Time to mature = 78

{week}

Planted leucaena area seedlings(t) = Planted leucaena area seedlings(t-1) + (Land allocation - Maturing)

INIT Planted leucaena area seedlings = 0

{ha}

INFLOWS:

Land allocation = (ARISA plantings +max(Smoothed planting decision, 0))*policy switch 1: shift to FL

{ha/week}

OUTFLOWS:

Maturing = delay(Land allocation, Time to mature)

{ha/week}

Graphical function (ARISA Planting):

Smoothed planting decision = SMTH3(Endog planting, 26)

{ha}

Endog planting = PULSE((Desired planted area-(Planted leucaena area mature trees+Planted leucaena area seedlings)), 45, 52)

{ha}

Desired planted area = IF Surplus feed CE>2 then 0 else MIN((Effect of profit on land allocation*Planted leucaena area mature trees), Total land)

{ha}

Surplus feed CE = Feedlot feeding capacity-(feedlot fat/22)

{cattle}

Feedlot feeding capacity = Total leucanea/Feed need per hd

{cattle}

Feed need per hd = 0.77

{tonne/cattle}

Total leucanea = Planted leucaena harvest volume+ Wild leucaena harvest volume

{tonne}

Planted leucaena harvest volume = (Planted leucaena area mature trees*Leucaena yield)/52

{tonnes}

Effect of profit on land allocation = IF Expected FL profit < 0 then 1 else (if time <208 then 1 else((Expected FL profit/init(Expected FL profit))^(Profit elasticity of land allocation)))

{Unitless}

Expected FL profit = DELAY3(FL profits, 104)

{Rp/week}

Profit elasticity of land allocation = 0.15

{unitless}

Total land(t) = Total land(t - 1)

INIT Total land = 5273

{ha}

**Wild leucaena**

Wild leucaena area(t) = wild leucaena area(t - 1)

INIT wild leucaena area = 75

{ha}

Wild leacaena production = (Wild leucaena yield*wild leucaena area/52)*policy switch 1: shift to FL

{tonne}

Wild leucaena harvest volume = (wild leacaena yield*Access to wild leucaena)

{tonne}

Graphical function (Access to wild leucaena):

Wild leucaena yield = 4

{tonne/ha}

Leucaena yield = 8.3

{tonnes/ha}

**GHG emission intensities**

Emission intensity = GHG emissions/total cattle LW

{GHG/kg/yr}

GHG emissions = project fatten pop*(47*25/52)+ Bull*47*25/52+Replace bull*47*25/52+(Weaners+ Calves)*47*25/52+(Heifer+ Pre adult female)*47*25/52+Replace female*47*25/52+Breeding cows*47*25/52

{total GHG/week}

Total cattle LW = (Bull selling*394+breed cow selling to butcher*260+selling breeders to II traders*210)*0.47+ (PF FL sales+Trad fat sales)*210*0.51

{kg}
