# Supplementary material for: Assessing the sustainable development and intensification potential of beef cattle production in Sumbawa, Indonesia, using a system dynamics approach
Source: PLoS One. 2017 Aug 17;12(8):e0183365. doi: 10.1371/journal.pone.0183365 (PMC5560717; doi:10.1371/journal.pone.0183365)
Supplement: S2 Appendix — (DOCX) [file pone.0183365.s003.docx]

## **S2 Appendix. Model validation tests**

In addition to the sensitivity analysis that was performed as part of the scale-out assessment, we performed a number of model validation tests recommended for SD models [9, 19]. First, we performed a Structure assessment, which requires that the model structure does not include any components, variables and parameters that do not exist in real life. Based on this assessment we found that the model structure is consistent with physical real-life system that the model represents. Second, we tested the Dimension consistency of the model, and we found that the model units were consistent and did not include any parameters without no real-life interpretation and meaning. Third, we performed a Structure-behavior test, which requires that the model generates logical behavior when a feedback loop is removed from the model. For example, when the feedback loop of lecuena planting (R2) is removed (i.e. setting acreage elasticity with respect to profit 0), the scaling-out intervention was not as successful as when lecuena production feedback loop was active. This is because removing that feedback loop removes the endogenous planting mechanism of leucena after exogenous project planting events complete. Finally, we conducted an Extreme condition test for the key model parameters. For example, forcing breeding stock to be zero (by disconnecting breeding stock from the rest of the production cycle) or forcing exogenous parameter constraints to extreme value generated expected and reasonable model results.
